# Yixintongmai Inhibits Proliferation and Migration and Promotes Apoptosis of Vascular Smooth Muscle Cells Cultured with High Glucose

**DOI:** 10.1155/2021/6583086

**Published:** 2021-05-04

**Authors:** Jingjing Guo, Di Zhao, Pingshuan Dong

**Affiliations:** ^1^Division of Hypertension, The First Affiliated Hospital, College of Clinical Medicine, Henan University of Science and Technology, Luoyang 471003, China; ^2^Division of Cardiology, The First Affiliated Hospital, College of Clinical Medicine, Henan University of Science and Technology, Luoyang 471003, China

## Abstract

**Objective:**

This study was designed to evaluate the effects of yixintongmai on proliferation, migration, and apoptosis of vascular smooth muscle cells (VSMCs) cultured with high glucose.

**Methods:**

VSMCs of the thoracic aorta from 5- to 8-week-old male Sprague-Dawley rats were cultured with normal (4.5 mM) or high (25 mM) glucose, respectively. The concentration of yixintongmai powder at 360 *μ*g/ml was chosen according to pre-experimental results.

**Results:**

Yixintongmai inhibited the proliferation of VSMCs (CCK-8 assay: 0.75 ± 0.04 versus 0.98 ± 0.09 OD, *P* < 0.001; cell counting: 37533 ± 1861 versus 56009 ± 3779 cells/well, *P* < 0.001) and the expression of proliferating cell nuclear antigen (0.74 ± 0.08 fold, *P* < 0.001) as compared with high glucose (HG). Yixintongmai inhibited the migration of VSMCs (transwell assay: 146 ± 16 versus 265 ± 62 cells; *P* < 0.001), scratch wound assay (0.17 ± 0.01 fold, *P* < 0.001), and the expression of matrix metalloproteinases-9 (0.87 ± 0.03 fold, *P* < 0.001) as compared with HG. Yixintongmai decreased mitochondrial membrane potentials (0.36 ± 0.12 fold, *P* < 0.001) and promoted early (2.11 ± 0.20 fold, *P* < 0.01) and late (2.11 ± 0.28 fold, *P* < 0.01) apoptosis of VSMCs as compared with HG. Yixintongmai inhibited the expression of B-cell lymphoma 2 (0.83 ± 0.07 fold, *P* < 0.01) and stimulated the activity of cleaved-capase-3/caspase-3 (2.00 ± 0.12 fold, *P* < 0.05) as compared with HG. Yixintongmai inhibited reactive oxygen species generation (0.46 ± 0.03 fold, *P* < 0.01) and the expression of NADPH oxidase-1 (0.84 ± 0.04 fold, *P* < 0.001), nuclear factor-kappa B (NF-*κ*B) p65 (0.71 ± 0.07 fold, *P* < 0.001), phosphorylated NF-*κ*B p65 (0.39 ± 0.02 fold, *P* < 0.0001), and inhibited nuclear translocation of NF-*κ*B p65 (0.87 ± 0.03 fold, *P* < 0.001) in VSMCs as compared with HG.

**Conclusions:**

Yixintongmai inhibits the proliferation and migration and promotes the apoptosis of VSMCs cultured with HG, which suggests the potential anti-atherosclerotic effects of this traditional Chinese medicine.

## 1. Introduction

Cardiovascular complications are the major cause of death in patients with type 2 diabetes mellitus (T2DM). The role of hyperglycemia and hyperinsulinemia in the pathogenesis of diabetic atherosclerosis is still largely unclear [1]. Chronic hyperglycemia disturbs the balance of vascular smooth muscle cells (VSMCs) between proliferation and apoptosis and facilitates migration from the media of vessel into the intima, which leads to neointimal hyperplasia and fibrous cap formation. Hyperglycemia-induced overproduction of reactive oxygen species (ROS) may be the key molecular mechanisms for diabetes mediated vascular damage [2]. ROS accumulation is critical for nuclear factor-kappa B (NF-*κ*B) activation. NF-*κ*B activation in VSMCs represents a key mechanism for the accelerated vascular disease observed in diabetes and is a pivotal stimulator for VSMCs dedifferentiation, proliferation, and migration [3]. Additionally, the increased activities of matrix metalloproteinases 2 (MMP-2) and MMP-9 play a role in extracellular matrix degradation thereby accelerating atherogenesis and potentially reducing plaque stability in diabetes [4].

Yixintongmai is a traditional Chinese medicine and is used in patients with coronary artery diseases in China. Yixintongmai consists of milkvetch root, ginseng, coastal glehnia root, figwort root, danshen root, sichuan lovage rhizome, turmeric root tuber, and prepared liquorice root. Several studies demonstrated that yixintongmai could inhibit restenosis after coronary angioplasty in patients with coronary artery diseases and T2DM [5]. Milkvetch root [6] and danshen root [7], the specific ingredient of yixintongmai, inhibited the progress of atherosclerosis through affecting the proliferation, migration, and apoptosis. Moreover, yixintongmai decreased the level of serum total cholesterol and triglycerides [5].

We hypothesized that yixintongmai regulated the proliferation, migration, and apoptosis of VSMCs. Therefore, this study was designed to evaluate the effects of yixintongmai on proliferation, migration, and apoptosis of VSMCs cultured with high glucose. Additionally, we determined the effects of yixintongmai on ROS generation and the expression of NF-*κ*B of VSMCs cultured with high glucose.

## 2. Methods

### 2.1. Reagents

The weight ratio of every component for 1000 g yixintongmai is as follows: 266 g (milkvetch root), 44 g (ginseng), 333 g (coastal glehnia root), 222 g (figwort root), 333 g (danshen root), 222 g (sichuan lovage rhizome), 222 g (turmeric root tuber), and 133 g (prepared liquorice root). Fetal bovine serum (FBS) was purchased from Biological Industries (Beit-Haemek, Israel). Dulbecco's Modified Eagle's Medium (DMEM) and phosphate buffer saline (PBS) were purchased from Hyclone Laboratories Inc (Logan, Utah, USA). Rabbit polyclonal antibody against NF-*κ*B p65, matrix metalloprotein 9 (MMP-9), NOX1 (NADPH1 oxidase-1), caspase-3, and mouse polyclonal antibody against proliferating cell nuclear antigen (PCNA) were purchased from Proteintech Group Inc. (Wuhan, China). Rabbit anti-mouse phosphorylated NF-*κ*B p65 was purchased from Santa Cruz Biotechnology (Santa Cruz, CA. USA). Secondary antibodies were purchased from CWBio (Beijing, China). Goat anti-Rabbit IgG (Alexa Fluor® 488) was purchased from Abcam (Cambridge, Lundon, England). Cell counting kit-8 and mitochondrial membrane potential detection kit was purchased from Solarbio Science & Technology (Beijing, China). N-acety-L-cysteine (NAC) 2′, 7′-dichlorofluorescindiacetate (DCFH-DA) was purchased from Beyotime Biotechnology (Shanghai, China). Collagenase-II was purchased from Biosharp (Nanshan, Guangdong, China). Transwell plates were purchased from Millipore (Bedford, MA, USA).

### 2.2. Animals

Male Sprague-Dawley rats (5-8-week-old) were purchased from HFK Bioscience Company (Beijing, China). All procedures were performed in accordance with the guidelines set by the Institutional Animal Care and Use Committee of the First Affiliated Hospital of Henan University of Science and Technology, which is in compliance with the Animal Research Reporting of In Vivo Experiments (ARRIVE) guidelines on animal research.

### 2.3. Cell Culture

VSMCs were prepared from the thoracic aorta of Sprague-Dawley rats. Whole thoracic aorta was isolated from sacrificed rats. The thoracic aorta was cut open longitudinally and the endothelial cells were removed with a sterile elbow tweezer scraping back and forth twice. The vascular adventitia was carefully stripped with ophthalmic tweezers and then was rinsed with phosphate-buffered saline (PBS) twice. The aortic tissue was cut into small pieces (1 mm^2^). Pieces of aortic tissue were digested with collagenase (125 U) for 2 h (shaking once every 30 min) at 37°C in a humidified 5% CO_2_ air atmosphere. When a great mass of cells split, five fold complete medium was added into the tube, and then the tube was centrifuged at 300g for 5 min. At last, cells were plated in T25 and were grown to confluence in DMEM with 5.5 mM glucose, 10% FBS, at 37°C in a humidified 5% CO_2_. Cells were grown to 80% confluences and then were used up to 8th [8].

### 2.4. Cell Treatment Protocol

VSMCs were divided into four groups. Control group (NG): DMEM medium with 5.5 mM D-glucose; high glucose group (HG): DMEM medium with 25 mM D-glucose; mannitol group (MG): DMEM medium with 5.5 mM D-glucose and 19.5 mM mannitol; yixintongmai group (HG + Y): DMEM medium with 25 mM D-glucose and 360 *μ*g/ml yixintongmai.

### 2.5. CCK-8 Assay

VSMCs proliferation was assayed with CCK-8 cell viability kit (Solarbio, Beijing, China). Firstly, VSMCs were randomly seeded at the density of 5 × 10^3^ cells/well and cultured in a 96-well plate. After cells reached 20–30% confluence, VSMCs were starved for 24 h with free serum DMEM medium and then incubated for 24 h to stimulate cell proliferation under different conditions as mentioned above. Subsequently, CCK-8 reagent (10 *μ*L) was added and cells were further incubated for 2 h at 37°C. The absorbance wavelength was read at 450 nm by spectrophotometric plate reader [9].

### 2.6. Western Blot

Total protein was extracted using an extraction kit (Solarbio, Beijing, China) according to the manufacturer's protocols, and the protein concentration was determined using a BCA protein assay kit (Solarbio, Beijing, China). Protein samples were separated by 12% sodium dodecyl sulfate polyacrylamide gel electrophoresis (40 mg per lane, 80 V, 2 h) and then transferred to polyvinylidene fluoride membranes (90 V, 120 min). After blocking with 5% non-fat dried milk in phosphate-buffered saline with Tween-20 (PBST) for 1 h, the membranes were separately incubated with the appropriate primary antibody, *β*-actin (1 : 5000), PCNA (1 : 3000), NF-*κ*B p65 (1 : 3000), phosphorylated NF-*κ*B p65(1 : 2000), MMP-9 (1 : 3000), caspase-3 (1 : 2000), and cleaved-caspase-3 (1 : 1500). They were then incubated with horseradish peroxidase-conjugated secondary antibody (Bioss, Beijing, China) and were visualized using the ECL Plus Detection Kit (Pierce Protein Research Products, Rockford, IL, USA). Protein expression was quantified by densitometry using ImageJ V1.8.0 (Bio-Rad, Hercules, CA, USA) with *β*-actin as an internal loading control.

### 2.7. Wound Healing Assay

Wound healing assay was used to evaluate the migration of VSMCs. VSMCs (4 × 10^4^ cells/well) were seeded and cultured in a six-well plate. After VSMCs grew to 80% confluences, VSMCs were incubated in serum-deprivation media for 24 h, and then a sterile plastic 1 ml micropipette tip was used to create a 1 mm scratch wound. Cells were continually cultured in different culture media as mentioned above. The scratched region was photographed immediately and at 48 h after scratching, and the migrating distance was thus calculated using Image Pro Plus 6.0 [10].

### 2.8. Transwell Assay

VSMCs migration was analyzed by Transwell assay. The cells were cultured in different culture media as mentioned above. After 24 h, cells were trypsinized with 0.25% (v/v) trypsin and re-suspended in the serum-free DMEM. Cells were counted and seeded in the upper chamber of each Transwell at the concentration of 1 × 10^5^ cells in 0.2 ml serum-free DMEM. 0.8 ml of DMEM supplemented with 20% FBS [11] was added to the lower chamber of each Transwell. Chambers were incubated for 12 h at 37°C with 5% CO_2_. Cells that migrated to the underside of the Transwell filter were fixed with 4% formaldehyde (w/v) for 20 min at room temperature and then stained with DAPI (1 : 10) for 10 min. The staining was examined by fluorescence microscopy at 200x magnification. The numbers of cells were calculated using Image Pro Plus 6.0.

### 2.9. Mitochondrial Membrane Potential Assay

VSMCs (4 × 10^5^ cells/well) were seeded in 6-well plates. After VSMCs grew to 30% confluence, VSMCs were incubated in serum-deprivation media for 24 h. And then the cells were cultured in different culture media as mentioned above. Subsequently, mitochondrial membrane potential was analyzed by the mitochondrial membrane potential assay kit using JC-1 according to manufacturer's instructions (Solarbio, Beijing, China).

### 2.10. Flow Cytometry

After starving with serum-free DMEM for 24 h, VSMCs were cultured in different culture media as mentioned above for 48 h and then were digested with 0.25% (v/v) trypsin. Then, cells were collected into centrifuge tubes (300x, 5 min) and were washed twice with PBS (4°C). Annexin-FITC/Propidium Iodide (PI) double staining (Solarbio, Beijing, China) was performed for cell apoptosis assessment. Firstly, VSMCs were resuspended in binding buffer (1.0 × 10^6^ cells/ml) and were added to new centrifuge tubes (100 *μ*l); Secondly, 5 *μ*l Annexin-FITC were added and reacted for 10 min in the absence of light. And then 5 *μ*l PI was added and reacted for 5 min in the absence of light. At last, 390 *μ*l PBS was added to centrifuge tubes and mixed uniform gently. BD Accuri C6 (Bio-Rad, Hercules, CA, USA) was executed to quantify the percentage of apoptotic cells.

### 2.11. Immunofluorescence

After starving with serum-free DMEM for 24 h, VSMCs were cultured in different culture media as mentioned above for 48 h. Then, VSMCs were washed three times with PBS and were incubated with ice-cold methanol for 10 min. After blocking non-specific binding sites with PBS containing 5% BSA, slides were stained with primary antibodies of rabbit anti-mouse NF-*κ*B p65 (1 : 100, 37°C, 3 h). Then, slides were washed with PBST and were incubated with goat anti-rabbit IgG (Alexa Fluor® 488) (1 : 500, 37°C, 1 h). Finally, nuclei were counterstained with DIPA (1 : 10, 10 min) and were washed three times with ultrapure water. Slides were examined in an OLYMPUS DP73 upright microscope (OLYMPUS, Tokyo, Japan). The examination wavelength was 543 nm (Green).

### 2.12. Measurements of Intracellular ROS

Intracellular ROS level was detected using the oxidant-sensitive probe DCFH-DA. VSMCs were seeded on six-well plates. After starving with serum-free DMEM for 24 h, the cells were cultured in different culture media as mentioned above for 48 h. And then cells were washed twice with PBS and were incubated with DCFH-DA at 5 *μ*mol/L for 30 min. The relative DCF fluorescence intensity was detected by fluorescent microscopy (Nikon, Tokyo, Japan). The examination wavelength was 488 nm and the emission wavelength was 530 nm, respectively.

### 2.13. Statistics Analysis

All statistical analysis of the data was performed using GraphPad Prism 6.0 software (GraphPad Software, San Diego, CA, USA). The results are presented as the means ± standard deviation (SD) of at least three independent experiments. One-way analysis of variance was used for statistical analysis of the data. In all cases, *P* values less than 0.05 were considered significant.

## 3. Results

### 3.1. Yixintongmai Inhibits the Proliferation of VSMCs and the Expression of PCNA of VSMCs Cultured with High Glucose

**Figure 1:**
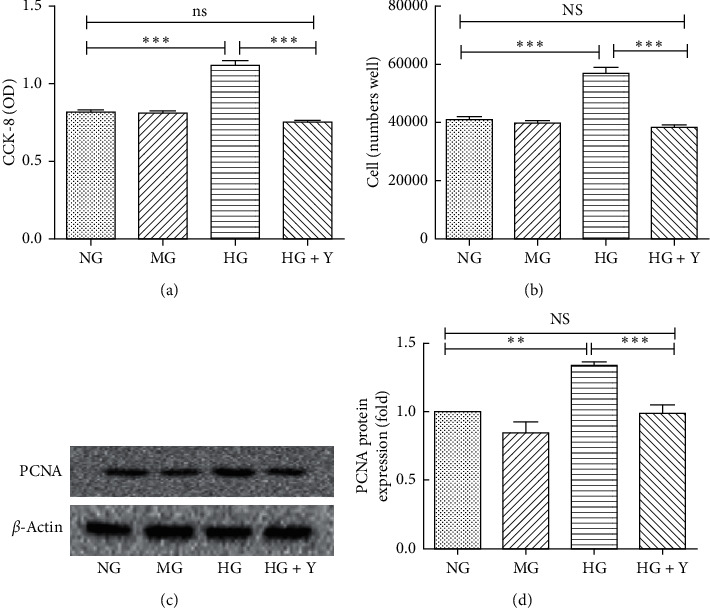
Effects of yixintongmai on proliferation and PCNA expression of VSMCs cultured with high glucose. NG: normal glucose (5.5 mM); MG: normal glucose (5.5 mM) and mannitol (19.5 mM); HG: high glucose (25 mM); HG + Y: high glucose (25 mM) and yixintongmai powder (360 *μ*g/ml); VSMCs proliferation were evaluated by CCK-8 (a) and cell counting (b); Western blot of PCNA expression (c) was quantified by densitometry using ImageJ V1.8.0 (d); densitometry quantification values are shown by fold over NG or HG. Mannitol was used as an osmolality control for high glucose condition. Values were expressed as means ± SD of sextuplicate experiments. ^*∗∗*^*P* < 0.01; ^*∗∗∗*^*P* < 0.001; NS: not significant (*P* > 0.05).

As shown in Figures 1(a) and 1(b)), yixintongmai inhibited the proliferation of VSMCs by CCK-8 (0.75 ± 0.04 versus 0.98 ± 0.09 OD, *P* < 0.001) and cell counting (37533 ± 1861 versus 56009 ± 3779 cells/well, *P* < 0.001) as compared with high glucose, respectively. As shown in Figures 1(c) and 1(d), yixintongmai significantly inhibited the expression of PCNA of VSMCs as compared with high glucose (0.74 ± 0.08 fold, *P* < 0.001).

### 3.2. Yixintongmai Inhibits the Migration of VSMCs and the Expression of MMP-9 of VSMCs Cultured with High Glucose

**Figure 2:**
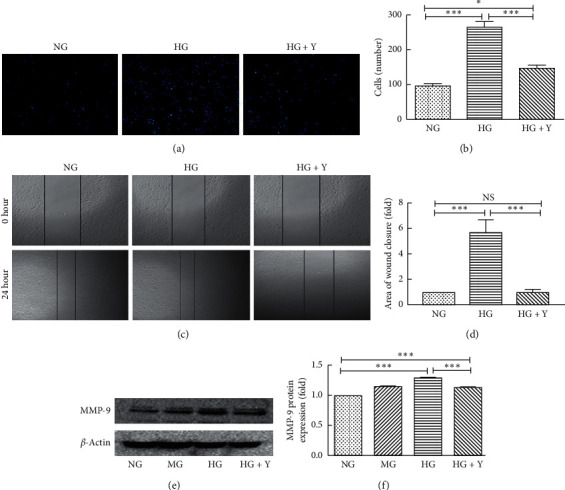
Effects of yixintongmai on migration and MMP-9 expression of VSMCs cultured with high glucose. NG: normal glucose (5.5 mM); HG: high glucose (25 mM); HG + Y: high glucose (25 mM) and yixintongmai powder (360 *μ*g/ml); MG: normal glucose (5.5 mM) and mannitol (19.5 mM). The migration of VSMCs was assessed using transwell assay (a, b) and scratch wound assay (c, d). In the transwell assay, the staining was examined by fluorescence microscopy at 100x magnification in three random fields. Fluorescent image (a); the mean numbers of VSMCs in three random fields (b); in wound healing assay, VSMCs migration was shown at 0 h and 24 h (c) and migration rate (d); Western blot of MMP-9 expression (e) was quantified by densitometry using ImageJ V1.8.0 (f). Densitometry quantification values are shown by fold over NG or HG. Values are expressed as means ± SD of triplicate experiments. ^*∗*^*P* < 0.05; ^*∗∗∗*^*P* < 0.001.

As shown in Figures 2(a) and 2(b), in transwell assay, yixintongmai significantly inhibited the migration of VSMCs as compared with high glucose (146 ± 16 versus 265 ± 62 cells, *P* < 0.001), whereas high glucose enhanced the ability of migration of VSMC as compared with normal glucose (265 ± 62 versus 95 ± 12 cells/well, *P* < 0.001). As shown in Figures 2(c) and 2(d), in scratch wound assay, yixintongmai significantly inhibited the migration of VSMCs as compared with high glucose (0.17 ± 0.01 fold, *P* < 0.001), whereas high glucose enhanced the migration of VSMCs as compared with normal glucose (5.7 ± 0.81 fold, *P* < 0.001). As shown in Figures 2(e) and 2(f), yixintongmai inhibited the expression of MMP-9 of VSMCs as compared with high glucose (0.87 ± 0.03 fold, *P* < 0.001), whereas high glucose enhanced the expression of MMP-9 as compared with normal glucose (1.27 ± 0.04 fold, *P* < 0.001).

### 3.3. Yixintongmai Promotes the Apoptosis and Inhibits the Expression of B-Cell Lymphoma 2 (Bcl-2) and the Activity of Caspase-3 of VSMCs Cultured with High Glucose

**Figure 3:**
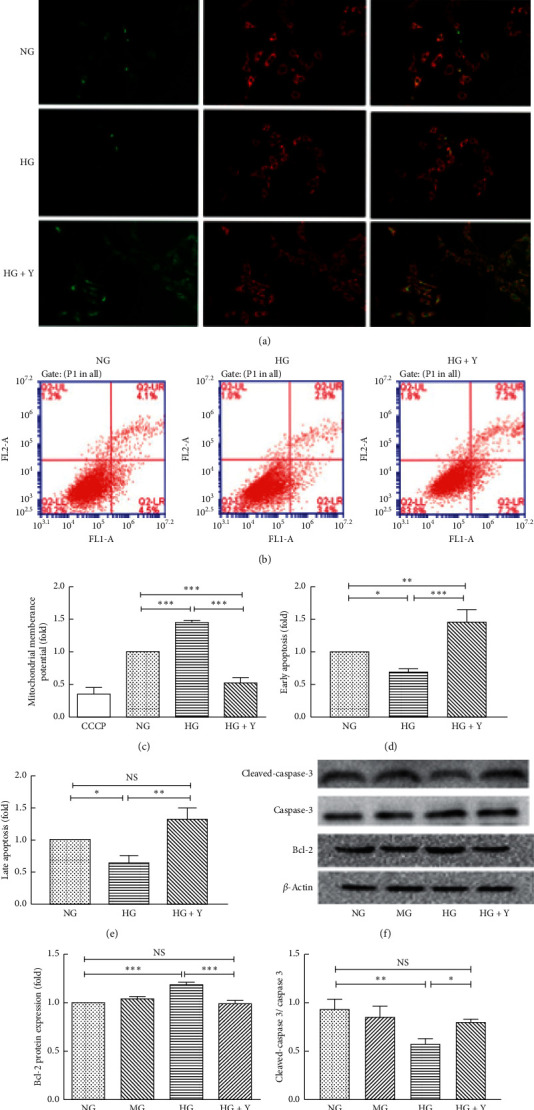
Yixintongmai promotes the apoptosis and inhibits the expression of Bcl-2 and the activity of caspase-3 of VSMCs cultured with high glucose. NG: normal glucose (5.5 mM); HG: high glucose (25 mM); HG + Y: high glucose (25 mM) and yixintongmai powder (360 *μ*g/ml); MG: normal glucose (5.5 mM) and mannitol (19.5 mM); Normal VSMCs stained with JC-1 emitted orange-red fluorescence with a little green fluorescence, reflecting the condition of mitochondrial membrane potentials. The increased ratio of orange-red fluorescence and green fluorescence indicates the elevation of mitochondrial membrane potentials, due to the increase of aggregated JC-1 within mitochondria. Fluorescence intensity in VSMCs was quantified using Image Pro Plus/IOD. The mitochondrial membrane potentials shown by the fluorescence intensity of VSMCs under different conditions (a); densitometry quantification of fluorescence intensity (c); flow cytometry Annexin V-FITC-PI assay in VSMCS in different groups (b, d, e). Lower right quadrant represents cells that undergo early apoptosis. Upper right quadrant represents cells that undergo late apoptosis. Western blot of Bcl-2 expression (f) and densitometry quantification (g); densitometry quantification values are shown by fold over NG or HG for each time point. Western bolt of caspase-3 and cleaved-caspase-3 expression (f), and densitometry quantification (h); the ratio of cleaved-caspase-3 and caspase-3 protein expression represented the activity of caspase-3. Values are expressed as means ± SD of triplicate experiments. ^*∗∗∗*^*P* < 0.001; ^*∗∗*^*P* < 0.01: ^*∗*^*P* < 0.05; NS: not significant (*P* > 0.05); CCCP: carbonyl cyano-to-chlorobenzene hydrazone (positive control).

As shown in Figures 3(a) and 3(c), high glucose increased mitochondrial membrane potentials of VSMCs as compared with normal glucose (1.45 ± 0.07 fold, *P* < 0.001), shown as the increased ratio of orange-red fluorescence and green fluorescence indicating the inhibition of apoptosis of VSMCs. Yixintongmai decreased mitochondrial membrane potentials of VSMCs as compared with high glucose (0.36 ± 0.12 fold, *P* < 0.001), indicating the promotion of apoptosis of VSMCs. As shown in Figures 3(b), 3(d), and 3(e), cells in the lower right quadrant were early apoptotic VSMCs, as the staining of these cells was Annexin^+^/PI^−^. High glucose inhibited early apoptosis of VSMCs as compared with normal glucose (0.69 ± 0.04 fold, *P* < 0.05). However, yixintongmai promoted early apoptosis of VSMCs as compared with high glucose (2.11 ± 0.20 fold, *P* < 0.01). Cells in the upper right quadrant were late apoptotic VSMCs, as the staining of these cells was Annexin^+^/PI^+^. High glucose inhibited late apoptosis of VSMCs as compared with normal glucose (0.62 ± 0.11 fold, *P* < 0.05). However, yixintongmai promoted late apoptosis of VSMCs as compared with high glucose (2.11 ± 0.28 fold, *P* < 0.01). As shown in Figures 3(f) and 3(g), yixintongmai inhibited the expression of Bcl-2 of VSMCs as compared with high glucose (0.83 ± 0.07 fold, *P* < 0.01), whereas high glucose stimulated the expression of Bcl-2 as compared with normal glucose (0.69 ± 0.04 fold, *P* < 0.01). As shown in Figures 3(f) and 3(h), yixintongmai stimulated the activity of caspase-3 of VSMCs cultured with high glucose (0.20 ± 0.12 fold, *P* < 0.001), whereas high glucose inhibited the activity of caspase-3 as compared with normal glucose (0.64 ± 0.04 fold, *P* < 0.05).

### 3.4. Yixintongmai Reduces Intracellular ROS Accumulation and the Expression of NOX1 of VSMCs Cultured with High Glucose

**Figure 4:**
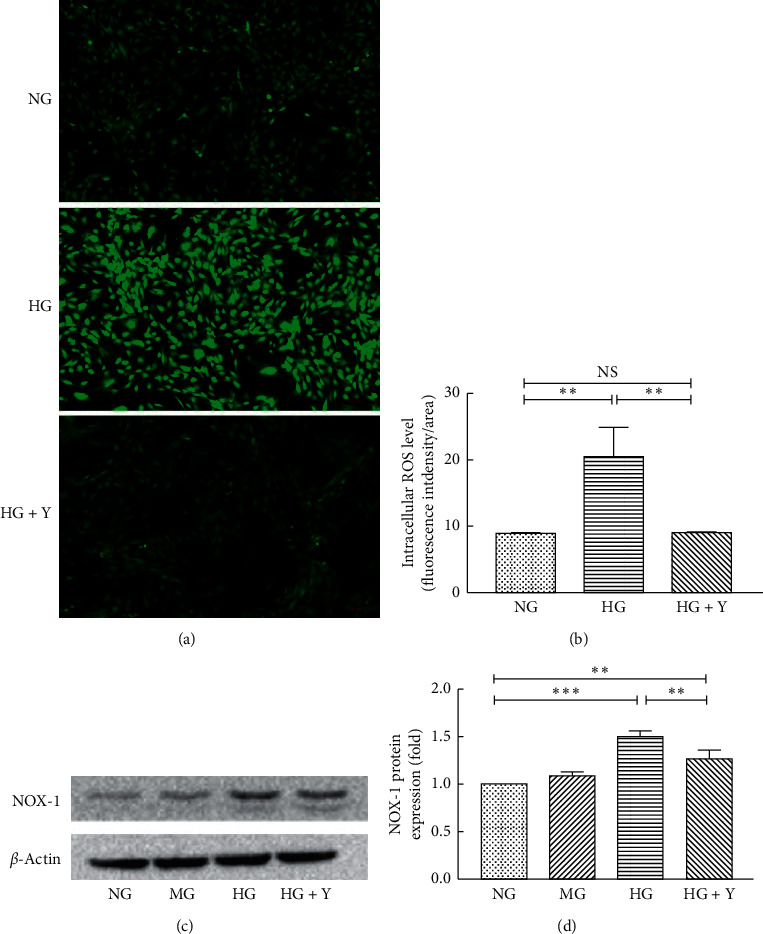
Yixintongmai inhibits ROS generation and expression of NOX-1 of VSMCs cultured with high glucose. NG: normal glucose (5.5 mM); HG: high glucose (25 mM); HG + Y: high glucose (25 mM) and yixintongmai powder (360 *μ*g/ml); MG: normal glucose (5.5 mM) and mannitol (19.5 mM). The intracellular ROS level shown by the fluorescence intensity of VSMCs under different conditions (a); densitometry quantification of mean fluorescence intensity values shown by the ratio of integrated density and area of VSMCs under different conditions (b); Western blot of expression of NOX-1 (c) and densitometry quantification (d); densitometry quantification values are shown by fold over NG or HG. Values are expressed as means ± SD of triplicate experiments. ^*∗*^*P* < 0.05; ^*∗∗∗*^*P* < 0.001; NS: not significant (*P* > 0.05).

As shown in Figures 4(a) and 4(b), yixintongmai inhibited ROS generation of VSMCs as compared with high glucose (0.46 ± 0.03 fold, *P* < 0.01), whereas high glucose promoted the level of intracellular ROS of VSMCs as compared with normal glucose (2.30 ± 0.45 fold, *P* < 0.01). As shown in Figures 4(c) and 4(d), yixintongmai inhibited the expression of NOX-1 of VSMCs as compared with high glucose (0.84 ± 0.04 fold, *P* < 0.001), whereas high glucose promoted the expression of NOX-1 of VSMCs as compared with normal glucose (1.50 ± 0.05 fold, *P* < 0.001).

### 3.5. Yixintongmai Inhibits Nuclear Translocation of NF-*κ*B and the Expression of NF-*κ*B of VSMCs Cultured with High Glucose

**Figure 5:**
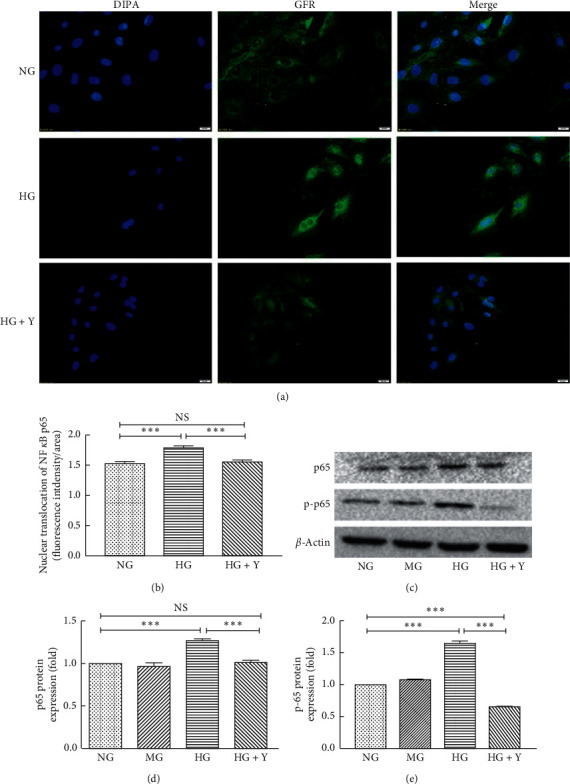
Yixintongmai inhibits nuclear translocation of NF-*κ*B and the expression of NF-*κ*B of VSMCs cultured with high glucose. NG: normal glucose (5.5 mM); HG: high glucose (25 mM); HG + Y: high glucose (25 mM) and yixintongmai powder (360 *μ*g/ml); the nuclear translocation of NF-*κ*B in VSMCs under different conditions (a) and densitometry quantification of mean fluorescence intensity values shown by the ratio of integrated density and area of VSMCs under different conditions (b); Western blot of the expression of NF-*κ*B p65 and phosphorylated NF-*κ*B p65 (c), and densitometry quantification (d, e). Densitometry quantification values are shown by fold over NG or HG. Values are expressed as means ± SD of triplicate experiments. ^*∗*^*P* < 0.05; ^*∗∗∗*^*P* < 0.001; NS: not significant (*P* > 0.05).

As shown in Figure 5, yixintongmai inhibited nuclear translocation of NF-*κ*B p65 (0.87 ± 0.03 fold, *P* < 0.001) (Figures 5(a) and 5(b)), the expression of NF-*κ*B p65 (0.71 ± 0.07 fold, *P* < 0.001), and phosphorylated NF-*κ*B p65 (0.39 ± 0.02 fold, *P* < 0.001) (Figures 5(c)–5(e)) as compared with high glucose, whereas high glucose promoted nuclear translocation of NF-*κ*B p65 (1.17 ± 0.03 fold, *P* < 0.001) (Figures 5(a) and 5(b)), the expression of NF-*κ*B p65 (1.27 ± 0.05 fold, *P* < 0.001), and phosphorylated NF-*κ*B p65 (1.65 ± 0.03 fold, *P* < 0.001) (Figures 5(c)–5(e)) in VSMCs as compared with normal glucose.

## 4. Discussion

In the present study, we provide the evidence that yixintongmai inhibits high glucose-induced VSMCs proliferation and migration and promotes apoptosis, suggesting the potential anti-atherosclerotic effects of this traditional Chinese medicine. Kobayashi et al. [12] reported that ligustilide had anti-proliferative effects on VSMCs. Yuan et al. [13] found that astragaloside IV, extracted from milkvetch root, rectified the imbalance of proliferation and apoptosis and regulated phenotypic modulation of VSMC induced by high glucose. N-butylidenephthalide, the active extraction of angelica sinensis, suppressed platelet aggregation and inhibited SMC proliferation [14]. Furthermore, danshen root inhibited thrombosis, reduced the level of serum lipids, and inhibited the formation of atherosclerotic plaque [15]. All these findings indicated that yixintongmai may have therapeutic potential in diabetes-associated cardiovascular diseases.

VSMCs proliferation is an early key event in the formation of atherosclerotic plaques. Milkvetch root-*Angelica* [16] and *Ligusticum* [17] inhibited VSMCs proliferation by decreasing the percentage of S and G2/M phase cells and increasing the number of cells in G0/G1 phases. Hiromu et al. [18] reported that PCNA, the pro-proliferation protein, regulated cell cycle progression from G1 phase to S phase. In this study, we found yixintongmai inhibited high glucose-induced VSMCs proliferation and the expression of PCNA, which suggested that yixintongmai inhibited high glucose-induced VSMCs proliferation via inhibiting cell cycle progression.

Upregulation of MMP-9 mediated the migration of VSMCs. ROS/NF*κ*B/MMP-9 signaling pathway may be involved in the migration of VSMCs [3]. Hyperglycemia increased intracellular formation of advanced glycosylation end products (AGEs) [1]. AGE, the receptor for AGE (RAGE) binding, activated the expression of MMP-9 [19]. Yan et al. [20] reported that Milkvetch root-*Angelica* combination inhibited the expression of MMP-9. In this study, yixintongmai inhibited the migration of VSMCs and the expression of MMP-9 induced by high glucose, suggesting the inhibition of MMP-9 expression may be one of the potential mechanisms of anti-atherosclerotic effects of yixintongmai.

Interestingly, yixintongmai promoted the apoptosis of VSMCs cultured with high glucose and inhibited the expression of Bcl-2 of VSMCs. The Bcl-2 protein expressed in VSMCs regulated the antioxidant pathway [21–23]. Bcl-2 could protect VSMCs against apoptosis [24]. Moreover, caspase-3 was considered as the primary executioner in the initiation of apoptosis [25]. Hyperglycemia inhibited the apoptosis of VSMCs through the upregulation of Bcl-2 family (Bcl-2, Bcl-xL, and Bfl-1) [24] and inhibited the activation of caspase-3 [26]. High glucose increased the expression of antiapoptotic proteins that may be important in the development of atherosclerosis in diabetic patients [27, 28]. Sara et al. [29] found that astragaloside and ligusticum downregulated the expression of Bcl-2. Shahzad et al. [30] reported that astragaloside IV and tetramethylpyrazine, one of the active ingredients of ligusticum, increased the expression of Bcl-2 in brain injury. Apoptosis was typically associated with loss of mitochondrial membrane polarization, leading to permeability changes and the release of Bcl-2 family [31]. Milkvetch root impaired mitochondrial function to trigger apoptosis of VSMCs [13]. Yixintongmai decreased mitochondrial membrane potential in this study. The loss of mitochondrial membrane permeability represented the occurrence of the membrane permeability transition. The effects of yixintongmai on expression of Bcl-2, the activity of caspase-3, and mitochondrial membrane permeability were coincident to these findings, which may mediate the effects of yixintongmai on apoptosis of VSMCs cultured with high glucose.

It is known that oxidative stress was commonly implicated as a major factor in the initiation and progression of diabetes-associated cardiovascular diseases [31]. Hyperglycemia could promote ROS accumulation [32], which were similar to our data. Juan et al. [33] reported that sichuan lovage rhizome effectively reduced the ROS formation, eliminated ROS to prevent lipid peroxidation, protected the mitochondrial function, and maintained mitochondrial membrane potential. As a potent ROS scavenger, danshen root inhibited high glucose-induced oxidative stress and reduced the generation of ROS and mitochondrial depolarization [34]. Polysaccharide of milkvetch root had antioxidant, antiviral activities and promoted pro-apoptosis [35]. These studies may partially explain the mechanisms of anti-atherosclerotic effects of yixintongmai. Bierhaus et al. found ROS overproduction induced by hyperglycemia activated the redox sensitive transcription factor NF-*κ*B [36]. A great deal of researches showed that NF-*κ*B activation was involved in proliferation, migration, inflammation, and oxidative stress of VSMCs [37, 38]. Lu et al. found that blockade of ROS generation diminished PDGF-induced NF-*κ*B p65 nuclear translocation, phosphorylation, and degradation of I-*κ*B. Phosphorylated NF-*κ*B p65 served a vital role in the activation of NF-*κ*B [37]. Chen et al. reported that sichuan lovage rhizome inhibited the expression of NF-*κ*B to accomplish anti-atherosclerosis effect [39]. Furthermore, both astragaloside IV and polysaccharides of milkvetch root inhibited phosphorylated NF-*κ*B p65 (see [40, 41]; p-p65). Our results confirmed the inhibitory effects of yixintongmai on the expression of NF-*κ*B p65 and nuclear translocation in VSMCs cultured in high glucose. NOX-1 in VSMCs had been described to be responsible for O2^.^- production and redox signaling in several pathological conditions including atherosclerosis, diabetes, and hypertension [42]. Salazar et al. reported that excess ROS production caused NF-*κ*B activation, which in turn upregulated NOX-1 expression inducing senescence of VSMCs [43]. Therefore, inhibition of ROS/NF-*κ*B signaling pathway may be one of anti-atherosclerotic mechanisms of yixintongmai.

In conclusion, yixintongmai inhibits the proliferation and migration and promotes the apoptosis of VSMCs cultured with high glucose, suggesting the potential anti-atherosclerotic effects of this traditional Chinese medicine. These effects may be mediated by inhibition of ROS/NF-*κ*B signal pathways.

## Data Availability

The data used to support the findings of this study are included within the article.
